# Beyond antibiotics: artificial intelligence–enabled anti-infective ecosystems for next-generation precision therapeutics against antimicrobial resistance

**DOI:** 10.3389/fcimb.2026.1897057

**Published:** 2026-08-26

**Authors:** Veilumuthu Pattapulavar, Sathiyabama Ramanujam, Rosshini Badmanathan, John Godwin Christopher

**Affiliations:** 1 Department of BioMedical Sciences, School of BioSciences and Technology, Vellore Institute of Technology (VIT), Vellore, Tamil Nadu, India; 2 Department of Physics, School of Physics, Madurai Kamaraj University, Madurai, Tamil Nadu, India

**Keywords:** antimicrobial peptides, antimicrobial resistance, artificial intelligence, bacteriophage therapy, CRISPR antimicrobials, microbiome therapeutics, nanotechnology, One Health

## Abstract

The rapid global expansion of antimicrobial resistance (AMR) threatens to undermine decades of progress in infectious disease management and highlights the limitations of conventional antibiotic-centered therapeutic strategies. Although emerging technologies—including antimicrobial peptides, bacteriophage therapy, CRISPR-based antimicrobials, microbiome therapeutics, anti-virulence approaches, nanotechnology-enabled drug delivery, and artificial intelligence (AI)—have individually demonstrated considerable promise, they are predominantly being developed as independent interventions rather than as coordinated components of an integrated therapeutic strategy. This Perspective proposes the Intelligent Anti-Infective Ecosystem (IAIE) as a conceptual systems-level framework that computationally integrates multimodal diagnostics, pathogen genomics, microbiome profiling, AI-assisted decision support, programmable precision therapeutics, ecological monitoring, and longitudinal clinical feedback within a continuously learning dynamically optimized workflow. Unlike existing paradigms that primarily optimize individual technologies or therapeutic decisions, IAIE emphasizes closed-loop coordination among complementary antimicrobial approaches to support precision-guided infection management while preserving microbiome integrity and mitigating resistance selection pressure. We further outline the core components, operational principles, translational challenges, and technology readiness of the major therapeutic platforms that could contribute to such an ecosystem, while distinguishing clinically established interventions from emerging experimental strategies. Importantly, IAIE should be interpreted as a prospective conceptual architecture rather than an existing clinical platform. Its proposed clinical value remains to be established through sequential computational, preclinical, and prospective clinical investigations using standardized microbiological, ecological, and patient-centered outcome measures. By framing antimicrobial innovation within an responsive systems perspective, IAIE provides a roadmap for future multidisciplinary research aimed at integrating artificial intelligence and systems microbiology to enable sustainable management of antimicrobial resistance.

## 1 Introduction

The discovery of penicillin revolutionized modern medicine and marked the beginning of the antibiotic era, transforming the treatment of infectious diseases and enabling advances in surgery, organ transplantation, intensive care, oncology, and numerous other medical disciplines. However, the remarkable success of antibiotics has been progressively undermined by the rapid emergence and global dissemination of antimicrobial resistance (AMR), which now represents one of the greatest threats to global public health (Henry, 2019). Multidrug-resistant pathogens increasingly compromise the effectiveness of existing antimicrobial therapies, resulting in prolonged hospitalization, escalating healthcare costs, and increased mortality. Recent global estimates indicate that bacterial AMR is already associated with millions of deaths annually and is projected to become one of the leading causes of mortality worldwide if effective interventions are not developed (Naghavi et al., 2024).

Importantly, the AMR crisis extends beyond the failure of individual antimicrobial agents. Rather, it reflects a complex evolutionary and ecological phenomenon driven by decades of antimicrobial use across human healthcare, veterinary medicine, agriculture, food production, and environmental systems (Flemming et al., 2016). Microorganisms continuously adapt through mutation, horizontal gene transfer, biofilm formation, and ecological selection, enabling the rapid emergence and dissemination of resistance determinants against nearly every major class of antibiotics (Davies and Davies, 2010). Simultaneously, the antimicrobial development pipeline has slowed considerably because of scientific, regulatory, and economic barriers that have limited the introduction of truly novel therapeutic classes (Lewis, 2020; Theuretzbacher et al., 2023).

Accordingly, antimicrobial resistance is now widely recognized as a One Health challenge that cannot be addressed solely within the boundaries of human medicine. Resistant microorganisms and antimicrobial resistance genes circulate continuously among humans, animals, food systems, and environmental reservoirs, creating interconnected transmission networks that require coordinated surveillance and intervention across multiple sectors (World Health Organization, 2015; FAO, UNEP, WHO and WOAH, 2022). This systems-level perspective highlights that sustainable antimicrobial stewardship must extend beyond antibiotic prescribing to include pathogen surveillance, microbiome preservation, environmental monitoring, and integrated public health strategies.

These challenges have accelerated the search for therapeutic approaches that move beyond conventional broad-spectrum microbial eradication toward precision-guided modulation of pathogens, host responses, and microbial ecosystems. Emerging technologies—including antimicrobial peptides, bacteriophage therapy (Sulakvelidze et al., 2001), CRISPR-based antimicrobials (Bikard et al., 2014), anti-virulence therapeutics, microbiome engineering (Belkaid and Hand, 2014), nanotechnology-enabled drug delivery, and artificial intelligence-assisted drug discovery—have each demonstrated considerable promise within their respective domains. Nevertheless, these technologies are generally investigated and developed as independent strategies despite addressing complementary aspects of infectious disease management.

Rather than introducing another therapeutic modality, we propose that the next major advance in anti-infective medicine may arise from the iterative integration of these complementary technologies within a unified systems framework. In this Perspective, we introduce the Intelligent Anti-Infective Ecosystem (IAIE) as a conceptual architecture that connects pathogen sensing, artificial intelligence-assisted prediction, programmable therapeutics, microbiome-informed ecological management, and continuous outcome monitoring through iterative feedback. The conceptual novelty of IAIE therefore lies not in any individual technology, but in the dynamic coordination of diagnostics, computational intelligence, therapeutic intervention, and ecological surveillance into a continuously learning decision-support framework. By integrating advances in artificial intelligence, systems microbiology, synthetic biology, targeted therapeutics, and One Health surveillance, IAIE provides a conceptual roadmap for developing dynamically optimized, ecosystem-oriented strategies to improve long-term management of antimicrobial resistance.

## 2 Why conventional antibiotics are failing

The progressive decline in antibiotic effectiveness is driven by the remarkable evolutionary adaptability of microbial pathogens. Conventional antibiotics primarily function by targeting essential cellular processes such as cell wall synthesis, protein translation, DNA replication, or metabolic pathways. Although highly effective initially, these mechanisms impose intense selective pressure that accelerates microbial evolution and resistance emergence (Lewis, 2020).

Bacteria rapidly acquire resistance through chromosomal mutations, plasmid-mediated gene transfer, transposons, integrons, and bacteriophage-mediated horizontal gene exchange (Davies and Davies, 2010). The widespread dissemination of resistance determinants such as carbapenemases, extended-spectrum β-lactamases, and colistin resistance genes demonstrates the extraordinary mobility of antimicrobial resistance across microbial populations and ecosystems. Modern healthcare environments further amplify resistance evolution through high antibiotic exposure, invasive procedures, and immunocompromised patient populations.

Biofilm formation represents another major limitation of conventional antimicrobial therapy. Biofilms are highly organized microbial communities encased within extracellular polymeric matrices that protect pathogens from antibiotics, host immune responses, and environmental stress (Flemming et al., 2016). Within biofilms, microorganisms exhibit altered metabolic states, reduced antibiotic penetration, enhanced genetic exchange, and increased persistence, making infections exceptionally difficult to eradicate. Chronic wound infections, catheter-associated infections, cystic fibrosis lung disease, and implant-associated infections frequently involve biofilm-mediated antimicrobial tolerance.

In addition to resistance evolution, conventional antibiotics often disrupt beneficial commensal microbiota that contribute fundamentally to immune regulation, colonization resistance, nutrient metabolism, and epithelial homeostasis (Blaser and Falkow, 2009). Antibiotic-induced dysbiosis can promote opportunistic infections, inflammatory disorders, metabolic dysfunction, and long-term ecological instability within the human microbiome (Lynch and Pedersen, 2016). The growing recognition of microbiome-associated health consequences has challenged the long-standing paradigm of broad-spectrum antimicrobial therapy.

Economic barriers have further contributed to the stagnation of antibiotic innovation. Antibiotic development is scientifically complex, financially risky, and commercially unattractive due to short treatment durations, rapid resistance emergence, and stewardship policies limiting widespread use. Consequently, many pharmaceutical companies have reduced investment in antibiotic research despite the escalating global AMR crisis (Theuretzbacher et al., 2023). These limitations indicate that simply developing stronger antibiotics may not provide a sustainable long-term solution. Instead, fundamentally different approaches that minimize evolutionary pressure, preserve microbial ecology, and selectively target pathogenic mechanisms are increasingly required.

## 3 Precision anti-pathogenic therapeutics

### 3.1 Antimicrobial peptides

Antimicrobial peptides (AMPs) are evolutionarily conserved components of innate immunity that exhibit broad-spectrum antimicrobial and host-defense activities (Cheng et al., 2024; Sarangi et al., 2026). Many AMPs exert antimicrobial activity through rapid physicochemical interactions with microbial membranes, leading to membrane destabilization and cell death. However, accumulating evidence indicates that AMP activity extends beyond membrane disruption and may also involve intracellular targets, including nucleic acids, proteins, and metabolic pathways (Islam et al., 2026; Gagandeep et al., 2024; Ma et al., 2024). Although the multimodal mechanisms of action and synergistic interactions observed among certain AMP combinations may increase the evolutionary complexity of resistance development, microbial adaptation and AMP resistance have nevertheless been documented. Therefore, AMPs should be viewed as promising but not resistance-proof therapeutic agents within next-generation anti-infective strategies (Medvedeva and Kolomeisky, 2026). In addition to their direct antimicrobial effects, AMPs can regulate inflammatory responses, recruit immune cells, enhance epithelial barrier function, and promote tissue repair, highlighting their multifunctional therapeutic potential (Hancock and Sahl, 2006). Recent advances in peptide engineering, synthetic biology, and computational design have enabled the development of optimized AMP candidates with improved stability, reduced toxicity, and enhanced therapeutic efficacy (Bahar and Ren, 2013). Furthermore, artificial intelligence-driven peptide discovery platforms are accelerating the identification of novel AMP sequences with activity against multidrug-resistant pathogens, expanding the potential of AMPs as key components of next-generation pathogen-selective therapeutics anti-infective systems (Mookherjee et al., 2020). Collectively, these advances position antimicrobial peptides as promising targeted therapeutics that combine direct antimicrobial activity with host-directed immunomodulatory functions. Continued improvements in computational peptide engineering and synthetic biology are expected to further enhance their therapeutic potential. Importantly, their compatibility with microbiome-preserving strategies creates opportunities for combination with other targeted antimicrobial approaches discussed in the following sections.

### 3.2 Bacteriophage therapy

Bacteriophages represent highly specific bacterial viruses capable of selectively infecting and eliminating bacterial pathogens while preserving commensal microbiota (Lin et al., 2017). Renewed interest in phage therapy has emerged due to increasing multidrug resistance and advances in phage engineering technologies (Zhang and Ahn, 2025).

Phages offer several advantages over conventional antibiotics, including strain-specific targeting, self-amplifying therapeutic activity, biofilm penetration, and reduced microbiome disruption. Recent compassionate-use clinical cases have demonstrated successful phage-mediated treatment of disseminated multidrug-resistant infections (Dedrick et al., 2019). Engineered bacteriophages capable of delivering biofilm-degrading enzymes, CRISPR payloads, or enhanced lytic functions have further expanded the therapeutic potential of phage-based systems. Phage therapy reflects a broader conceptual shift toward ecological targeted medicine in which pathogens are selectively targeted without indiscriminate microbial elimination. Recent advances further extend the therapeutic scope of bacteriophages beyond bacterial eradication. Their ability to selectively deliver genetic payloads and biofilm-targeting enzymes provides a logical interface with programmable antimicrobial technologies, particularly CRISPR-based systems, which are discussed in the following section.

### 3.3 CRISPR-based antimicrobials

CRISPR-Cas Systems have emerged as one of the most transformative platforms in next-generation antimicrobial development. Unlike traditional antibiotics, CRISPR-based therapeutics enable sequence-specific targeting of pathogenic bacteria, resistance genes, and virulence determinants at the genetic level (Barrangou and Marraffini, 2014).

CRISPR antimicrobials can selectively eliminate resistant strains while preserving beneficial microbiota, representing a major advancement in pathogen-selective therapeutics anti-infective medicine (Bikard et al., 2014). Potential applications include targeted removal of antimicrobial resistance genes, programmable microbiome editing, suppression of virulence pathways, and prevention of horizontal gene transfer (Citorik et al., 2014).

The integration of CRISPR technologies with bacteriophage delivery systems, nanocarriers, and artificial intelligence-guided target prediction may ultimately enable dynamically optimized and personalized antimicrobial therapeutics tailored to individual pathogen profiles and microbiome compositions (Hille et al., 2018). Although considerable translational challenges remain, CRISPR technologies substantially expand the targeted of antimicrobial intervention by enabling programmable manipulation of pathogen genomes and resistance determinants. Their greatest future potential may arise when combined with complementary therapeutic approaches that reduce pathogenic fitness while minimizing ecological disruption.

### 3.4 Anti-virulence therapeutics

Anti-virulence therapy represents a fundamentally different strategy that focuses on disarming pathogens rather than directly killing them (Clatworthy et al., 2007). These approaches target virulence determinants including toxins, quorum-sensing networks, secretion systems, adhesion molecules, and biofilm-associated mechanisms (Rasko and Sperandio, 2010). Because anti-virulence approaches target pathogenic behaviors rather than directly inhibiting microbial growth, they have been proposed as strategies that may reduce selective pressure for resistance compared with conventional bactericidal antibiotics. However, this advantage should not be considered universal, as resistance and continuously updated responses may still arise when virulence-associated pathways contribute to microbial fitness, ecological competitiveness, or host colonization. Recent studies on quorum-sensing inhibitors have highlighted both their therapeutic promise and the potential for evolutionary adaptation, underscoring the need for careful long-term evaluation of resistance dynamics (Alum et al., 2025; Papaneophytou, 2026). Quorum-sensing inhibitors, for example, disrupt microbial communication pathways regulating coordinated virulence expression and biofilm formation (Ng and Bassler, 2009). Targeting pathogenic behavior rather than microbial viability aligns closely with emerging ecological models of infection management and offers important opportunities for microbiome preservation and resistance mitigation. Rather than replacing conventional antimicrobial therapy, anti-virulence strategies broaden the therapeutic repertoire by selectively attenuating pathogenicity while preserving ecological balance. This ecological perspective naturally aligns with emerging microbiome-based therapeutic approaches that seek to restore microbial homeostasis rather than eradicate microbial communities indiscriminately.

### 3.5 Microbiome therapeutics

Microbiome Therapeutics have rapidly emerged as a major frontier in anti-infective medicine. Rather than eliminating microorganisms indiscriminately, microbiome-targeted therapies aim to restore ecological balance, reinforce colonization resistance, and support host immune homeostasis (Buffie and Pamer, 2013). Approaches including probiotics, prebiotics, synbiotics, fecal microbiota transplantation, and engineered live biotherapeutic products have demonstrated promising therapeutic potential in infectious and inflammatory diseases. Fecal microbiota transplantation has demonstrated remarkable efficacy against recurrent Clostridioides difficile infection (van Nood et al., 2013). Advances in synthetic biology now allow engineering of programmable microbial therapeutics capable of sensing pathogens, delivering antimicrobials, modulating immunity, and dynamically responding to disease-associated signals. The microbiome paradigm reframes infectious disease management as an ecological system challenge rather than a purely pathogen-centric problem (Charbonneau et al., 2020). Consequently, microbiome engineering functions as the ecological stabilization layer of IAIEs, supporting long-term resilience against pathogen colonization and resistance emergence. Collectively, these advances shift infectious disease management from pathogen elimination toward ecological restoration. As microbiome-directed therapies continue to mature, their integration with precision delivery technologies may enable increasingly targeted and context-dependent antimicrobial interventions.

### 3.6 Nanotechnology-driven therapeutics

Nanotechnology has transformed the design of next-generation anti-infective platforms through enhanced targeting pathogen-selective therapeutics, controlled drug release, biofilm penetration, and multimodal antimicrobial mechanisms (Huang et al., 2024; Serrano et al., 2025). Nanoparticles exert antimicrobial activity through membrane disruption, oxidative stress generation, intracellular interference, and enhanced drug delivery efficiency. Smart nanomaterials responsive to environmental stimuli such as pH, enzymes, or temperature can enable highly selective antimicrobial release within infection microenvironments (Hajipour et al., 2012). Integration of nanotechnology with CRISPR systems, phage therapy, biosensors, and artificial intelligence-guided design may ultimately enable intelligent antimicrobial nanoplatforms capable of dynamically optimized therapeutic responses. As a result, nanotechnology serves as an enabling delivery platform capable of integrating CRISPR systems, antimicrobial peptides, bacteriophages, biosensors, and AI-guided therapeutic control into a unified antimicrobial ecosystem. Nanotechnology provides versatile delivery strategies capable of improving targeting specificity, controlled release, and therapeutic stability across multiple antimicrobial modalities. These advances also create opportunities for integrating diagnostic information, responsive drug delivery, and computational decision support, thereby establishing an important technological foundation for the systems-level framework discussed in the following section. To illustrate the conceptual contribution of this Perspective, **Table 1** compares conventional antibiotic therapy with the proposed Intelligent Anti-Infective Ecosystem (IAIE) framework across key therapeutic dimensions, including biological strategy, target specificity, adaptive capability, technology readiness, and future clinical direction. Rather than comparing individual technologies, the table highlights the systems-level transition from conventional antimicrobial treatment toward an integrated, AI-enabled precision anti-infective ecosystem.

**Table 1:** Conceptual transition from conventional antibiotics to intelligent anti-infective ecosystems.

Therapeutic dimension	Conventional antibiotics	Intelligent anti-infective ecosystems	Strategic advantage against AMR	Representative evidence
Therapeutic philosophy	Broad-spectrum microbial eradication	Precision ecological modulation of pathogens and microbial communities	Reduced collateral microbiome disruption and sustainable infection control	(Clatworthy et al., 2007; Rasko and Sperandio, 2010)
Target specificity	Non-selective killing of pathogens and commensals	Pathogen-selective targeting and programmable antimicrobial activity	Lower selective pressure and preservation of beneficial microbiota	(Bikard et al., 2014; Citorik et al., 2014)
Resistance evolution	Rapid resistance emergence through continuous selective pressure	Adaptive and precision-guided pathogen suppression	Slower resistance development and improved therapeutic durability	(Davies, 2011; Lewis, 2020)
Biofilm management	Poor penetration and reduced efficacy in mature biofilms	Active biofilm disruption and targeted eradication	Enhanced treatment of persistent and chronic infections	(Flemming et al., 2016; Chang et al., 2022)
Microbiome impact	Dysbiosis and ecological instability	Microbiome preservation and restoration	Improved immune homeostasis and colonization resistance	(Blaser and Falkow, 2009; Buffie and Pamer, 2013)
Antimicrobial mechanism	Direct inhibition of essential bacterial growth pathways	Virulence suppression, gene editing, ecological modulation, and immune enhancement	Reduced evolutionary pressure on microbial survival	(Ng and Bassler, 2009; LaSarre and Federle, 2013)
Drug discovery strategy	Empirical and time-intensive screening approaches	Artificial intelligence-driven predictive antimicrobial discovery	Accelerated identification of novel therapeutics	(Lewis, 2020; Stokes et al., 2020)
Delivery systems	Systemic and non-specific drug exposure	Smart targeted and responsive delivery platforms	Enhanced therapeutic precision and reduced toxicity	(Hajipour et al., 2012)
Therapeutic adaptability	Static chemical therapeutics	Programmable and dynamically adaptive therapeutic systems	Real-time response to evolving pathogens	(Hille et al., 2018; Charbonneau et al., 2020)
Core enabling technologies	Conventional medicinal chemistry	AI, CRISPR, synthetic biology, phage engineering, microbiome therapeutics, nanotechnology	Multidimensional precision and precision anti-infective medicine	(Dedrick et al., 2019; Stokes et al., 2020)
Future clinical direction	Development of stronger broad-spectrum antibiotics	Personalized intelligent anti-infective ecosystem medicine	Sustainable long-term management of AMR	(Naghavi et al., 2024)
Current translational status	Clinically established standard-of-care	Components are at different stages of maturity: fecal microbiota transplantation is clinically established for selected indications; bacteriophage therapy has encouraging early clinical evidence; AI is increasingly used for antimicrobial discovery and clinical decision support; nanotechnology has partial clinical translation; CRISPR-based antimicrobials, engineered microbiome therapeutics, and fully integrated IAIE platforms remain predominantly preclinical or proof-of-concept.	Clarifies technology readiness, translational priorities, and realistic expectations for future implementation	(van Nood et al., 2013; Dedrick et al., 2019)

AI, artificial intelligence; CRISPR, clustered regularly interspaced short palindromic repeats; AMR, antimicrobial resistance; IAIE, Intelligent Anti-Infective Ecosystem.

## 4 Artificial intelligence as the integrative engine of intelligent anti-infective medicine

Artificial intelligence (AI) has emerged as an important enabling technology for next-generation antimicrobial discovery and targeted infection management. Early demonstrations of AI-assisted antibiotic discovery, including the identification of novel antibacterial compounds using deep learning, illustrated the potential of machine learning to accelerate therapeutic discovery beyond conventional screening approaches (Stokes et al., 2020). More recently, advances in protein language models and structural prediction algorithms, including AlphaFold and AlphaFold 3, have substantially improved the inference of protein structures, protein–ligand interactions, and sequence–structure relationships from biological sequence data (Jumper et al., 2021; Abramson et al., 2024).

Within antimicrobial research, these computational approaches have primarily been applied to antimicrobial peptide engineering, structural characterization of bioactive molecules, therapeutic target prioritization, and rational optimization of candidate compounds rather than complete end-to-end antimicrobial development pipelines. For example, studies integrating AlphaFold-based structural prediction with molecular dynamics simulations and computational peptide design have improved prediction of antimicrobial peptide activity, stability, toxicity, and structural properties, thereby facilitating candidate prioritization for subsequent experimental validation (Anand et al., 2025; Islam et al., 2026). Nevertheless, comprehensive antimicrobial discovery workflows integrating protein language models, structural prediction, experimental optimization, and clinical validation remain at an early stage of development. Accordingly, these computational platforms should presently be regarded as enabling technologies that accelerate hypothesis generation, molecular design, and candidate prioritization rather than independently validated antimicrobial development systems. Beyond accelerating antimicrobial discovery, AI has the potential to integrate diverse biological and clinical data streams into coordinated decision-support workflows. Emerging applications include pathogen genome analysis, antimicrobial resistance prediction, microbiome data interpretation, therapeutic optimization, and clinical decision support, each of which has demonstrated encouraging progress within its respective domain (Stokes et al., 2020; Jumper et al., 2021; Abramson et al., 2024). However, these capabilities currently exist largely as independent computational approaches, and no comprehensive AI platform has yet integrated pathogen genomic surveillance, microbiome profiling, therapeutic optimization, ecological monitoring, and longitudinal clinical feedback into a unified operational anti-infective system. Consequently, the Intelligent Anti-Infective Ecosystem (IAIE) proposed in this Perspective should be interpreted as a conceptual systems architecture rather than an existing technological platform. Within this framework, artificial intelligence is conceptualized as a future systems-level orchestration layer that could coordinate multiple evidence streams to support dynamically optimized therapeutic decision-making through continuous learning and iterative optimization. The feasibility, clinical utility, and implementation of such integrated systems remain to be established through rigorous multidisciplinary research involving computational biology, systems medicine, digital health infrastructure, and prospective clinical validation.

## 5 Convergence of technologies: toward intelligent anti-infective systems

Intelligent Anti-Infective Ecosystem (IAIE) as responsive, closed-loop systems architecture for infectious disease management in which pathogen sensing, artificial intelligence-assisted prediction, programmable therapeutic intervention, ecological monitoring, and iterative learning operate as interconnected components of a continuously evolving therapeutic network. Unlike conventional approaches that combine multiple antimicrobial technologies in parallel, IAIE emphasizes dynamic interactions among these components through continuous feedback, enabling therapeutic strategies to be refined in response to pathogen evolution, host characteristics, microbiome dynamics, and longitudinal clinical outcomes.

### 5.1 Intelligent anti-infective ecosystems

The rapid emergence of artificial intelligence (AI), programmable antimicrobials, microbiome engineering, bacteriophage therapy, nanotechnology-enabled drug delivery, and synthetic biology has substantially expanded the repertoire of pathogen-selective therapeutics anti-infective strategies. Although these technologies have often been discussed collectively as complementary innovations, they are generally developed and evaluated as independent therapeutic modalities. Consequently, current convergence models primarily describe the coexistence of multiple technologies rather than defining how they should interact within a unified therapeutic system.

To address this conceptual gap, we propose the Intelligent Anti-Infective Ecosystem as responsive, closed-loop systems framework for infectious disease management in which pathogen sensing, computational prediction, programmable therapeutic intervention, ecological monitoring, and iterative clinical learning function as interconnected components of a continuously evolving therapeutic network. Unlike conventional antimicrobial paradigms that primarily focus on selecting individual therapeutic agents, IAIE continuously integrates pathogen genomics, host-specific clinical characteristics, microbiome dynamics, antimicrobial resistance surveillance, and longitudinal therapeutic outcomes through feedback-driven optimization. Consequently, therapeutic strategies are not considered static treatment decisions but dynamically optimized processes that evolve in response to changing biological and clinical conditions (Dedrick et al., 2019).

The conceptual novelty of IAIE does not arise from introducing new antimicrobial technologies, nor simply from aggregating existing therapeutic approaches within a single framework. Rather, IAIE defines the organizational principles through which existing technologies interact dynamically to generate dynamically optimized therapeutic decision-making. Within this framework, AI functions as the computational coordination layer that integrates multidimensional biological and clinical information to support pathogen characterization, resistance prediction, therapeutic prioritization, ecological monitoring, and continuous refinement of intervention strategies. The emphasis therefore shifts from individual technological performance to system-level coordination among diagnostics, prediction, intervention, and feedback (Charbonneau et al., 2020).

Accordingly, IAIE should be regarded as a conceptual systems architecture rather than a catalogue of emerging antimicrobial technologies. Its defining characteristic is the establishment of continuous feedback loops linking pathogen evolution, host responses, microbiome ecology, therapeutic performance, and computational learning into an integrated decision-support framework. These interconnected processes generate emergent responsive properties that cannot be achieved when individual therapeutic modalities are implemented independently. By emphasizing continuous learning and dynamic therapeutic optimization, IAIE represents a conceptual transition from static antimicrobial treatment toward adaptive ecosystem-oriented infection management capable of responding to the evolving complexity of antimicrobial resistance (Bikard et al., 2014). The conceptual novelty of IAIE therefore does not arise from any individual therapeutic technology, nor simply from their coexistence. Rather, it emerges from the establishment of a continuously learning therapeutic ecosystem in which computational intelligence integrates diagnostic information, predicts resistance trajectories, supports therapeutic selection, monitors ecological consequences, and iteratively updates intervention strategies over time. Accordingly, IAIE should be viewed as a systems-level organizational framework rather than as a collection of emerging antimicrobial technologies.

### 5.2 Operational architecture of intelligent anti-infective ecosystems

The operational realization of the Intelligent Anti-Infective Ecosystem (IAIE) relies on the coordinated interaction of complementary technologies through continuous computational feedback rather than the independent application of individual therapeutic modalities. Although recent studies have highlighted the convergence of artificial intelligence (AI), synthetic biology, microbiome engineering, biomaterials, and targeted antimicrobial strategies, these approaches largely describe technological coexistence rather than a unified Intelligent Anti-Infective framework (Guo et al., 2024; Jiang et al., 2026). Building upon these advances, IAIE proposes a systems-level architecture in which diagnostics, computational intelligence, programmable therapeutics, ecological monitoring, and longitudinal clinical learning operate as an integrated and continuously network rather than as isolated technologies.

Future validation of the IAIE framework should follow a staged translational pathway encompassing computational modelling, preclinical investigation, and prospective clinical evaluation. Initial studies may integrate pathogen genomic sequencing, antimicrobial susceptibility profiles, microbiome composition, host clinical metadata, and longitudinal treatment outcomes to evaluate AI-assisted therapeutic optimization through computational simulations. Subsequently, preclinical infection models may investigate coordinated deployment of programmable antimicrobials, bacteriophage therapy, antimicrobial peptides, microbiome-directed interventions, and nanotechnology-enabled drug delivery systems. Ultimately, prospective randomized clinical trials will be required to determine whether IAIE-guided therapeutic management provides measurable clinical advantages compared with contemporary standard-of-care antimicrobial treatment.

Recent reviews have increasingly emphasized the convergence of emerging antimicrobial technologies as a promising strategy for addressing the growing complexity of antimicrobial resistance. For example, Guo et al. reviewed advances in bacterial engineering and synthetic biology for targeted therapeutic applications, highlighting how engineered microbial systems may enable highly targeted therapeutic interventions through programmable biological functions. Likewise, Jiang et al. summarized the expanding role of antimicrobial peptides within microbiome-aware therapeutic strategies, emphasizing ecological preservation and host–microbiome homeostasis as critical considerations for future anti-infective development. Together, these studies demonstrate an important transition from conventional pathogen eradication toward more integrated, adaptive anti-infective ecosystem intervention strategies that simultaneously consider pathogen biology, host responses, and microbial ecology.

The IAIE workflow begins with comprehensive characterization of both the pathogen and the host through rapid molecular diagnostics, pathogen whole-genome sequencing, antimicrobial susceptibility profiling, microbiome analysis, host clinical metadata, and, where appropriate, One Health surveillance information. Integration of these multidimensional datasets enables evidence-informed therapeutic planning that extends beyond conventional pathogen identification toward a systems-level understanding of infection biology. Such comprehensive data integration forms the foundation for pathogen-selective therapeutics anti-infective decision-making and reflects the increasing role of AI-assisted analytics in infectious disease management (Stokes et al., 2020).

Artificial intelligence serves as the computational coordination layer of IAIE by integrating heterogeneous biological and clinical information into clinically interpretable decision support. Rather than functioning as an autonomous therapeutic system, AI is envisioned as an evidence-based decision-support platform that assists clinicians by predicting antimicrobial resistance, prioritizing therapeutic options, forecasting potential ecological consequences, and supporting individualized treatment optimization. Importantly, the proposed framework does not imply that a fully integrated AI-driven anti-infective platform currently exists in clinical practice. Instead, IAIE represents a conceptual architecture describing how existing computational tools could be systematically integrated with emerging targeted antimicrobial technologies in future translational research and clinical implementation.

Following computational analysis, AI-assisted recommendations guide the rational deployment of complementary targeted antimicrobial interventions according to the biological characteristics of each infection. CRISPR-based antimicrobials offer programmable elimination of resistance determinants or virulence-associated genes, whereas bacteriophage therapy provides pathogen-selective bacterial killing with minimal disruption of beneficial microbiota (Bikard et al., 2014; Dedrick et al., 2019). Antimicrobial peptides contribute additional therapeutic flexibility through multiple mechanisms of microbial killing and immunomodulation, while anti-virulence therapeutics attenuate bacterial pathogenicity without necessarily imposing the intense selective pressure associated with conventional bactericidal antibiotics. Nanotechnology-enabled drug delivery systems may further improve therapeutic stability, targeted tissue distribution, controlled drug release, and biofilm penetration, thereby enhancing the effectiveness of multiple antimicrobial modalities while minimizing systemic toxicity.

Beyond direct pathogen elimination, IAIE explicitly incorporates microbiome engineering as a central therapeutic objective rather than a secondary consequence of treatment. Strategies including targeted probiotics, postbiotics, engineered microbial therapeutics, microbiome restoration, and ecological modulation may preserve microbial community resilience during antimicrobial therapy while facilitating recovery following infection (Charbonneau et al., 2020) (Charbonneau et al., 2020). Recent reviews have further emphasized the importance of microbiome-aware therapeutic strategies and ecological antimicrobial interventions as essential components of future targeted medicine (Jiang et al., 2026). Within the IAIE framework, preservation of microbiome integrity therefore becomes a measurable therapeutic endpoint alongside pathogen clearance, clinical recovery, and resistance containment.

A defining characteristic of IAIE is the incorporation of continuous feedback mechanisms that distinguish the framework from conventional antimicrobial treatment paradigms. Following therapeutic intervention, longitudinal assessment of pathogen evolution, antimicrobial susceptibility, microbiome composition, host immune responses, adverse events, and clinical outcomes generates updated information that is continuously re-integrated into the computational decision-support system. This iterative learning process enables therapeutic recommendations to be refined throughout treatment while simultaneously improving future decision-making through cumulative clinical experience. Consequently, infection management evolves from a sequence of isolated therapeutic decisions into a continuously learning Intelligent Anti-Infective Ecosystems capable of responding to changing biological and clinical conditions.

**Figure 1:**
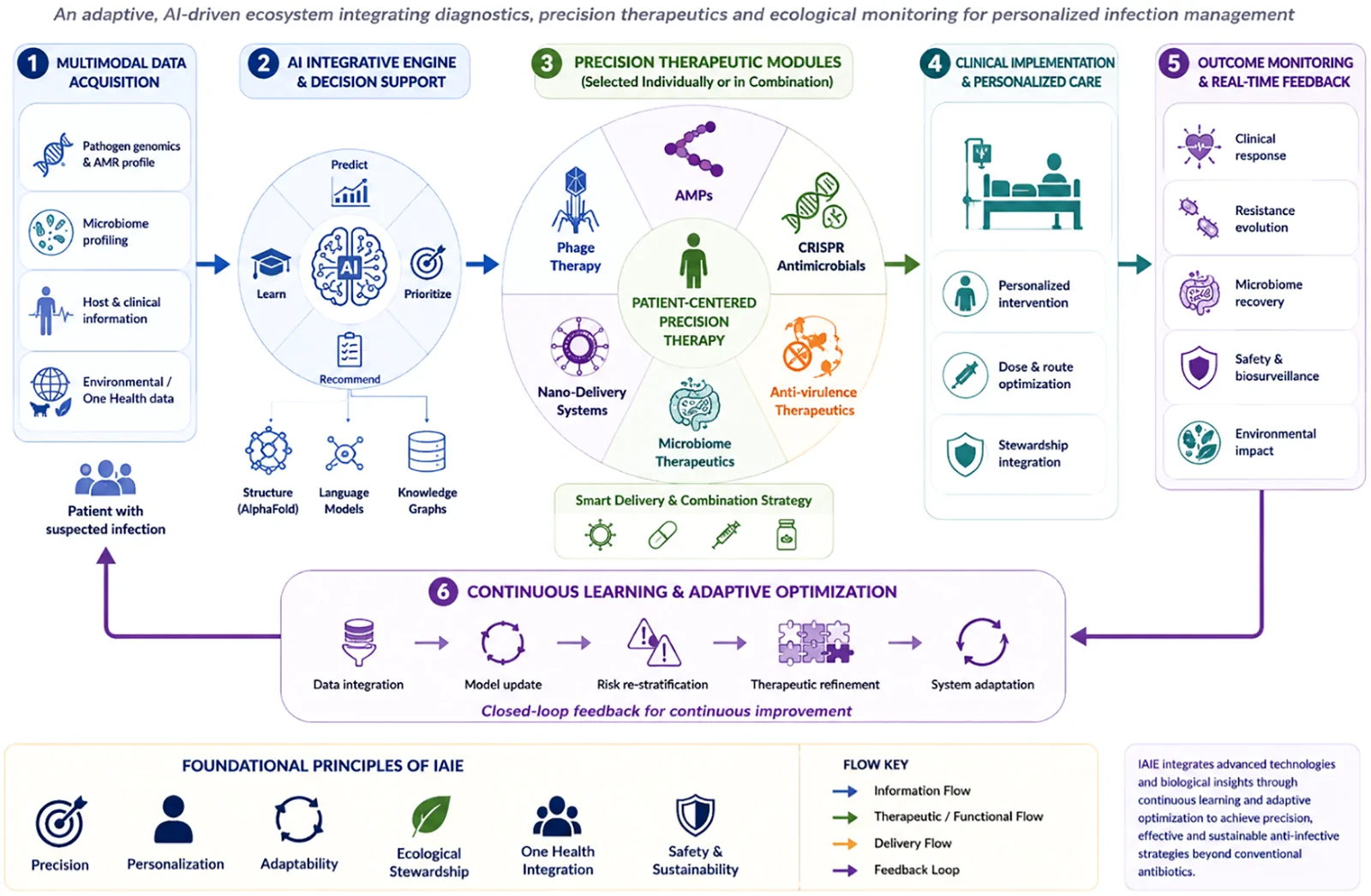
Conceptual workflow of the proposed Intelligent Anti-Infective Ecosystem (IAIE). The framework illustrates how multimodal clinical, microbiological, microbiome, and One Health surveillance data are integrated through an AI-assisted decision-support engine to guide targeted therapeutic selection, longitudinal monitoring, and continuous learning. The workflow represents a conceptual systems architecture proposed for future investigation rather than an existing clinically implemented platform.

Importantly, IAIE should be interpreted as a forward-looking conceptual framework rather than an established clinical platform. Many of its individual components—including AI-assisted diagnostics, CRISPR-based antimicrobials, bacteriophage therapy, microbiome therapeutics, antimicrobial peptides, and nanotechnology-enabled drug delivery—remain at different stages of technological and clinical maturity. Nevertheless, their coordinated integration provides a rational roadmap for developing next-generation pathogen-selective therapeutics anti-infective systems capable of simultaneously optimizing therapeutic efficacy, preserving microbiome integrity, mitigating antimicrobial resistance, and supporting sustainable infection management. **Figure 1** summarizes the proposed operational architecture of IAIE as an dynamically optimized, closed-loop framework linking diagnostics, computational prediction, targeted therapeutics, ecological monitoring, and continuous learning. **Figure 1** illustrates the proposed IAIE as a conceptual systems architecture rather than an implementation-specific engineering blueprint. The framework is intended to highlight the functional relationships among diagnostics, computational intelligence targeted therapeutics, ecological monitoring, and iterative clinical learning that collectively support infectious disease management. The figure therefore represents an organizational model for future research and clinical translation rather than a fully operational computational platform.

The individual components illustrated in **Figure 1** should not be interpreted as fixed sequential modules. Rather, they represent interconnected functional domains that continuously exchange biological, clinical, and ecological information through iterative feedback. The purpose of this conceptual representation is to illustrate the organizational principles underlying IAIE rather than to prescribe a specific software architecture, computational workflow, or engineering implementation. The **Table 2** summarizes conceptual distinctions between IAIE and existing infectious disease management frameworks. It is intended to compare organizational principles rather than implementation-specific technical architectures.

**Table 2:** Comparison of intelligent anti-infective ecosystems (IAIE) with existing frameworks for infectious disease management.

Framework	Primary objective	Core Components	Decision-making strategy	Feedback & learning	Ecological (microbiome) integration	Major limitation	Advancement introduced by IAIE
Conventional antimicrobial therapy (O'Neill, 2016)	Pathogen eradication	Broad-spectrum or targeted antibiotics	Static treatment selection based primarily on diagnosis and susceptibility testing	None	Limited consideration	High selective pressure leading to antimicrobial resistance and microbiome disruption	IAIE shifts from static pathogen eradication toward responsive, ecosystem-oriented therapeutic management.
Precision medicine (Ashley, 2016)	Individualized patient treatment	Host genomics, biomarkers, clinical characteristics	Personalized therapy selection	Generally limited to initial treatment selection	Limited	Primarily patient-centric and largely static after therapy initiation	IAIE continuously refines therapeutic strategies by integrating pathogen evolution, microbiome dynamics, and longitudinal clinical outcomes.
One Health (FAO, UNEP, WHO, and WOAH, 2022)	Integrated surveillance across human, animal, and environmental health	Epidemiological surveillance, environmental monitoring, public health interventions	Population-level risk assessment	Limited therapeutic feedback	Indirect	Strong surveillance framework but limited direct therapeutic integration	IAIE incorporates One Health surveillance directly into therapeutic decision-making through computational feedback.
AI-enabled antimicrobial discovery (Stokes et al., 2020; Yang et al., 2025)	Discovery and optimization of antimicrobial compounds	Machine learning, deep learning, molecular prediction	AI-assisted compound identification	Model refinement during drug development	Minimal	Primarily focused on drug discovery rather than clinical treatment optimization	IAIE extends AI beyond discovery by coordinating diagnostics, therapeutic selection, resistance prediction, ecological monitoring, and continuous clinical learning.
Digital health/Clinical decision support systems (Topol, 2019; Zhao et al., 2023)	Improve clinical decision-making	Electronic health records, predictive analytics, clinical decision support	Data-driven clinical recommendations	Variable	Minimal	Usually disconnected from programmable antimicrobial interventions	IAIE integrates clinical decision support with targeted therapeutics, microbiome preservation, and dynamically optimized therapeutic optimization.
Integrated bacterial engineering and microbiome-directed therapies (Guo et al., 2024; Jiang et al., 2026)	Precision microbial interventions	Engineered bacteria, antimicrobial peptides, microbiome modulation, synthetic biology	Technology-specific implementation	Limited	High	Focused on individual therapeutic platforms without systems-level adaptive coordination	IAIE integrates multiple targeted antimicrobial modalities into a unified computational framework with continuous feedback and iterative learning.
Intelligent Anti-Infective Ecosystem (IAIE) (This Perspective)	Adaptive precision infection management while minimizing antimicrobial resistance and preserving microbiome integrity	AI, pathogen genomics, microbiome profiling, CRISPR antimicrobials, bacteriophages, antimicrobial peptides, anti-virulence therapeutics, nanotechnology-enabled delivery systems, ecological monitoring, One Health surveillance	AI-assisted therapeutic responsive optimization through continuous systems-level integration	Continuous closed-loop learning using longitudinal biological and clinical feedback	Core therapeutic objective	Conceptual framework requiring experimental and clinical validation	Introduces a computationally coordinated, adaptive systems architecture that continuously integrates diagnostics, prediction, targeted therapeutics, ecological monitoring, and longitudinal learning to optimize infection management.

### 5.3 Distinguishing intelligent anti-infective ecosystems from existing frameworks

Although recent advances in artificial intelligence (AI), systems microbiology, synthetic biology, microbiome engineering, bacteriophage therapy, antimicrobial peptides, and nanotechnology have substantially expanded the landscape of precision anti-infective research, most existing frameworks remain technology-centric rather than systems-centric. Current approaches generally focus on optimizing individual components of infectious disease management, including AI-assisted antimicrobial discovery, personalized treatment through targeted medicine, population-level surveillance under the One Health framework, or digital clinical decision-support systems (Ashley, 2016; Topol, 2019; Stokes et al., 2020). While these approaches have significantly advanced antimicrobial research and clinical practice, they primarily function as parallel innovations with limited integration into a continuously responsive therapeutic framework.

The growing convergence of artificial intelligence, synthetic biology, microbiome engineering, biomaterials, programmable antimicrobials, and ecological medicine has recently been recognized in several comprehensive reviews. Guo et al. highlighted the therapeutic potential of engineered bacterial systems for precision medicine, whereas Jiang et al. emphasized antimicrobial peptides as integral components of microbiome-aware intervention strategies that preserve ecological balance while combating antimicrobial resistance. Similarly, recent advances in artificial intelligence-assisted antimicrobial discovery and intelligent healthcare architectures have demonstrated the increasing capability of computational methods to accelerate therapeutic discovery and support clinical decision-making. Collectively, these studies provide an important scientific foundation for next-generation antimicrobial innovation; however, they primarily focus on the development or convergence of individual technologies rather than defining an dynamically optimized systems architecture capable of continuously coordinating therapeutic decision-making through computational feedback.

Recent reviews have increasingly emphasized the convergence of emerging antimicrobial technologies. For example, Guo et al. highlighted bacterial engineering and synthetic biological strategies as promising platforms for targeted therapeutics, whereas Jiang et al. discussed antimicrobial peptides within broader microbiome-aware intervention strategies, emphasizing the importance of ecological considerations in future antimicrobial development (Guo et al., 2024; Jiang et al., 2026). Similarly, advances in AI-assisted antimicrobial discovery and intelligent healthcare architectures have demonstrated the g rowing potential of computational methods to support antimicrobial research and clinical decision-making (Stokes et al., 2020; Zhao et al., 2023). Collectively, these studies establish an important scientific foundation for next-generation antimicrobial innovation; however, they primarily describe complementary technological advances rather than an dynamically optimized systems architecture capable of continuously coordinating therapeutic decision-making.

The IAIE framework intentionally emphasizes functional integration over implementation detail. Accordingly, the proposed architecture should be interpreted as a conceptual organizational model describing how existing precision anti-infective technologies may interact within a continuously learning therapeutic ecosystem. Future computational implementation, data standards, interoperability, and software engineering will require dedicated translational research beyond the scope of the present Perspective.

The proposed Intelligent Anti-Infective Ecosystem (IAIE) extends these existing concepts by introducing a systems-level organizational framework in which diagnostics, computational prediction, programmable therapeutics, ecological monitoring, and longitudinal clinical outcomes are interconnected through continuous feedback loops. Rather than viewing AI, CRISPR-based antimicrobials, bacteriophage therapy, antimicrobial peptides, microbiome engineering, anti-virulence strategies, and nanotechnology-enabled drug delivery as independent therapeutic platforms, IAIE conceptualizes these technologies as complementary functional modules operating within a unified responsive network. Artificial intelligence serves as the computational coordination layer that continuously integrates pathogen genomics, microbiome dynamics, antimicrobial susceptibility profiles, host-specific clinical characteristics, and therapeutic outcomes to support iterative refinement of treatment strategies.

A distinguishing feature of IAIE is the generation of emergent systems-level properties that arise from coordinated interaction among its components rather than from the individual technologies themselves. Continuous integration of pathogen evolution, host responses, microbiome ecology, ecological surveillance, and therapeutic performance enables dynamically optimized therapeutic optimization, anticipatory resistance management, preservation of microbiome homeostasis, and progressive improvement of clinical decision support through iterative learning. These emergent properties differentiate IAIE from conventional precision medicine, One Health, digital health, and AI-enabled antimicrobial stewardship frameworks, which generally emphasize individualized treatment, surveillance, data management, or antimicrobial discovery independently rather than their continuous computational coordination (Ashley, 2016; FAO, UNEP, WHO, and WOAH, 2022; Topol, 2019). Accordingly, the conceptual contribution of the present Perspective is not to introduce additional therapeutic technologies but to propose an organizational framework through which these complementary advances may function as components of a continuously learning anti-infective ecosystem.

Importantly, IAIE is not intended to replace existing antimicrobial paradigms but rather to provide an integrative conceptual framework capable of harmonizing these complementary advances into a continuously learning therapeutic ecosystem. Accordingly, the principal contribution of this Perspective is not the introduction of novel antimicrobial technologies, but the proposal of an responsive systems architecture that computationally coordinates existing precision anti-infective strategies to improve therapeutic sustainability, antimicrobial stewardship, and long-term management of antimicrobial resistance.

Importantly, the IAIE framework should not be interpreted as suggesting that integration alone eliminates the intrinsic limitations of individual antimicrobial modalities. Antimicrobial peptides may still be susceptible to resistance development, anti-virulence therapies may exert selective pressures under specific ecological conditions, bacteriophage therapy faces challenges related to host range and regulatory standardization, CRISPR-based antimicrobials require efficient and safe delivery systems, microbiome-directed interventions remain highly individualized, and nanotechnology-based platforms continue to face translational and manufacturing barriers. The conceptual value of IAIE therefore lies not in overcoming these limitations completely, but in providing an Intelligent Anti-Infective framework through which complementary therapeutic strategies may collectively improve robustness, flexibility, and long-term infection management.

### 5.4 Conceptual proposition and future validation

Although substantial advances have been achieved in artificial intelligence, systems microbiology, synthetic biology, microbiome engineering, targeted antimicrobials, and digital health, current antimicrobial management frameworks remain largely technology-centric rather than systems-centric. Existing paradigms—including targeted medicine, antimicrobial stewardship, One Health surveillance, and AI-assisted clinical decision support—have significantly improved specific aspects of infectious disease management but generally function as parallel innovations with limited continuous interaction throughout the therapeutic process (Ashley, 2016; Topol, 2019; WHO et al., 2022). This Perspective proposes the Intelligent Anti-Infective Ecosystem (IAIE) as a conceptual systems architecture that computationally coordinates these complementary approaches within a continuously learning therapeutic framework.

Unlike existing models that optimize individual therapeutic components, IAIE is designed as a closed-loop dynamically optimized ecosystem in which pathogen genomics, antimicrobial resistance profiling, microbiome characterization, host clinical information, ecological surveillance, artificial intelligence-assisted prediction, programmable therapeutics, and longitudinal patient outcomes are continuously integrated through iterative feedback. Within this framework, therapeutic decisions are not viewed as isolated interventions but as dynamic processes that can be refined as new microbiological, ecological, and clinical information becomes available.

The central hypothesis of IAIE is that responsive integration of complementary targeted antimicrobial technologies may provide more sustainable long-term management of antimicrobial resistance than conventional static antimicrobial strategies. Specifically, we propose that coordinated use of pathogen genomic surveillance, microbiome-informed therapeutic selection, AI-assisted decision support, programmable antimicrobial interventions, ecological monitoring, and continuous clinical learning could reduce resistance emergence while preserving microbiome integrity and improving therapeutic durability. However, this proposition should be interpreted as a conceptual hypothesis requiring rigorous validation, rather than as an established clinical conclusion.

Future validation should follow a staged translational pathway. Initial investigations may employ computational modelling using integrated pathogen genomic datasets, antimicrobial susceptibility profiles, microbiome sequencing data, pharmacological information, and longitudinal clinical metadata to evaluate AI-assisted therapeutic optimization. Subsequent preclinical studies should examine coordinated implementation of antimicrobial peptides, bacteriophage therapy, CRISPR-based antimicrobials, microbiome-directed interventions, and nanotechnology-enabled delivery systems in appropriate infection models. Ultimately, prospective randomized clinical studies will be required to determine whether IAIE-guided therapeutic management provides measurable advantages over current standards of care.

To facilitate rigorous evaluation, comparator groups should include conventional antimicrobial therapy, established antimicrobial stewardship programmes, and AI-assisted decision-support systems that do not incorporate continuous ecological feedback. Consistent with recent antimicrobial clinical investigations and pharmacovigilance studies (Zhuang et al., 2023; Yang et al., 2025; Zhao et al., 2026), predefined outcome measures should include microbiological eradication, clinical cure, emergence of antimicrobial resistance, recurrence of infection, microbiome recovery, treatment-related adverse events, antimicrobial utilization, and longer-term patient outcomes.

Importantly, the technologies incorporated within IAIE remain at different stages of scientific and clinical maturity. While microbiome therapeutics have achieved clinical implementation in selected indications and AI-assisted decision support is increasingly being adopted in healthcare, engineered bacteriophages, CRISPR-based antimicrobials, programmable antimicrobial peptides, and integrated dynamically optimized therapeutic platforms remain largely at the preclinical or early translational stage. Consequently, IAIE should be regarded as a long-term translational roadmap rather than an immediately deployable clinical platform.

Successful implementation will additionally require standardized multi-omics data acquisition, interoperable computational infrastructure, explainable artificial intelligence, prospective model validation, regulatory harmonization, cybersecurity, ethical governance, and close collaboration among clinicians, microbiologists, computational scientists, engineers, and regulatory authorities. These scientific and regulatory challenges reinforce that the proposed framework represents a future research direction rather than a mature healthcare system.

ccordingly, IAIE should not be interpreted as a replacement for precision medicine, One Health, antimicrobial stewardship, or digital health. Instead, it provides a conceptual organizational architecture capable of computationally coordinating these complementary frameworks within a continuously learning anti-infective ecosystem. The principal contribution of this Perspective therefore lies not in introducing new antimicrobial technologies, but in proposing a systems-level framework through which existing and emerging precision antimicrobial strategies may eventually operate as coordinated components of an dynamically optimized therapeutic ecosystem.

## 6 Translational challenges and regulatory bottlenecks

Despite their transformative potential, next-generation anti-pathogenic technologies face substantial scientific, regulatory, manufacturing, and implementation barriers before widespread clinical adoption can be achieved. Unlike conventional small-molecule antibiotics, many emerging antimicrobial platforms are iterative personalized, biologically complex, and continuously evolving, creating unique challenges for regulatory evaluation and long-term risk assessment. Bacteriophage therapy faces significant translational hurdles related to manufacturing standardization, batch-to-batch consistency, host-range characterization, quality control, and regulatory classification. The personalized nature of phage cocktails may challenge conventional drug approval pathways developed for fixed pharmaceutical products. Regulatory agencies including the FDA and EMA continue to evaluate appropriate frameworks for assessing phage-based therapeutics while balancing safety, efficacy, and manufacturing flexibility (Food and Drug Administration (FDA), 2025 and European Medicines Agency (EMA), 2024). CRISPR-based antimicrobials introduce additional biosafety, ecological, and ethical considerations. Potential off-target genetic modifications, horizontal gene transfer, environmental dissemination of engineered genetic elements, and unintended ecological consequences require comprehensive risk assessment and long-term monitoring strategies (Teffera et al., 2026; Wizrah, 2026).

Establishing standardized containment protocols, environmental surveillance systems, and internationally harmonized governance mechanisms will be essential before large-scale clinical deployment. Microbiome-based therapeutics present distinct challenges associated with donor variability, ecosystem complexity, long-term ecological stability, and patient-specific responses. Because microbiome interventions intentionally alter complex microbial communities, conventional short-term efficacy endpoints may be insufficient. Future regulatory evaluation may require longitudinal monitoring of microbiome composition, ecological resilience, resistance gene dynamics, and host physiological outcomes to establish durable safety and effectiveness. Nanotechnology-enabled antimicrobial systems similarly require extensive assessment of biodistribution, pharmacokinetics, tissue accumulation, immunogenicity, environmental persistence, and long-term toxicity. Nanomaterials may exhibit biological behaviors that differ substantially from traditional pharmaceuticals, necessitating specialized testing frameworks capable of evaluating both therapeutic efficacy and ecological safety (DeFilipp et al., 2019).

Artificial intelligence–enabled antimicrobial platforms present an additional layer of regulatory complexity. Clinical implementation requires transparent algorithms, reproducible computational workflows, high-quality training datasets, explainable decision-making processes, and continuous post-deployment validation. Regulatory agencies increasingly recognize that AI systems may evolve over time through dynamically optimized learning processes, raising important questions regarding accountability, bias mitigation, model drift, cybersecurity, and clinical oversight. Successful implementation of Intelligent Anti-Infective Ecosystems (IAIEs) will therefore require coordinated efforts among microbiologists, clinicians, computational scientists, bioengineers, public-health agencies, industry stakeholders, and policymakers. International organizations such as the World Health Organization (WHO, 2015), alongside regulatory authorities including the FDA and EMA, will play critical roles in establishing globally harmonized standards for safety assessment, clinical validation, antimicrobial stewardship, environmental monitoring, and responsible deployment. Ultimately, the clinical translation of Intelligent Anti-Infective Ecosystems will depend not only on technological innovation but also on the development of robust regulatory infrastructures capable of evaluating highly dynamic, personalized, and ecosystem-oriented therapeutic platforms.

### 6.1 Clinical readiness and technology maturity

Although the technologies discussed in this Perspective collectively illustrate the future direction of anti-infective therapeutics, they differ substantially in their stage of translational development. Microbiome-based interventions, including fecal microbiota transplantation for recurrent Clostridioides difficile infection, have already demonstrated clinical efficacy and are incorporated into selected clinical practice guidelines (van Nood et al., 2013. Likewise, several antimicrobial peptides and nanotechnology-enabled formulations have progressed into clinical evaluation, although relatively few have achieved routine clinical use owing to challenges related to toxicity, pharmacokinetics, manufacturing scalability, and regulatory approval (Mookherjee et al., 2020; Serrano et al., 2025). Bacteriophage therapy has shown encouraging outcomes in compassionate-use cases and early clinical studies, particularly for multidrug-resistant bacterial infections, yet standardized regulatory pathways, phage manufacturing, and large randomized clinical trials remain limited (Dedrick et al., 2019). In contrast, CRISPR-based antimicrobials, programmable microbiome engineering, and AI-guided dynamically optimized therapeutic systems remain largely at the experimental or preclinical stage despite rapid technological progress (Bikard et al., 2014; Citorik et al., 2014; Stokes et al., 2020). Accordingly, the IAIE framework should not be interpreted as representing technologies with uniform clinical maturity. Rather, it provides a conceptual roadmap illustrating how interventions at different stages of development may ultimately be integrated through computational decision support and continuous learning as individual technologies mature. Future progress will require coordinated advances in clinical validation, regulatory science, manufacturing, digital health infrastructure, biosafety assessment, and multidisciplinary collaboration. The technologies incorporated within IAIE span different stages of translational development, ranging from clinically established interventions to proof-of-concept experimental platforms. Recognizing these differences is essential for realistic interpretation of the proposed framework. Microbiome-based therapeutics, particularly fecal microbiota transplantation for recurrent Clostridioides difficile infection, already possess established clinical evidence and regulatory approval in selected indications, whereas engineered live biotherapeutic products remain under active clinical investigation (van Nood et al., 2013). Bacteriophage therapy has demonstrated encouraging outcomes in compassionate-use cases and early clinical studies but has not yet achieved widespread regulatory standardization (Dedrick et al., 2019). Artificial intelligence has already become an important tool for antimicrobial discovery, molecular design, and clinical decision support, although comprehensive AI-guided dynamically optimized therapeutic ecosystems have not yet been clinically implemented (Stokes et al., 2020). Likewise, CRISPR-based antimicrobials, programmable microbiome engineering, and integrated Intelligent Anti-Infective Ecosystem (IAIE) platforms remain predominantly at the experimental or proof-of-concept stage. Recognizing these differences in technology readiness is essential for distinguishing currently deployable therapeutic approaches from longer-term research directions.

## 7 Future outlook: toward intelligent anti-infective ecosystems

The post-antibiotic era will likely be defined not by the discovery of a single “super antibiotic,” but by the emergence of intelligent, adaptive, and ecosystem-oriented therapeutic systems capable of selectively modulating microbial behavior and host–microbe interactions.

Future infectious disease management may increasingly involve personalized anti-infective therapies guided by real-time pathogen genomics, microbiome profiling, AI-driven predictive analytics, and targeted therapeutic engineering. Infection control strategies may transition from reactive treatment toward predictive microbial ecosystem management integrating One Health principles across human, animal, and environmental systems. Emerging advances in digital microbiology, synthetic biology, and computational medicine are progressively redefining the conceptual boundaries of antimicrobial therapy (O'Neill, 2016).

Instead of indiscriminate pathogen eradication, future therapeutics may focus on ecological stabilization, virulence suppression, microbiome restoration, and responsive pathogen containment. Such a transformation represents not merely a technological evolution but a fundamental paradigm shift in modern medicine. Intelligent anti-infective systems (IAIEs) should be developed within a One Health framework that recognizes the interconnected nature of antimicrobial resistance across human, animal, agricultural, and environmental ecosystems. Resistance determinants circulate continuously among clinical pathogens, livestock-associated microbiota, wastewater networks, and environmental reservoirs, creating globally interconnected resistance pathways. Consequently, sustainable antimicrobial strategies will require integrated surveillance systems, pathogen-selective therapeutics stewardship programs, environmentally responsible therapeutic design, and coordinated cross-sectoral interventions. The convergence of artificial intelligence, genomic epidemiology, systems microbiology, and ecological monitoring may ultimately enable predictive antimicrobial management strategies operating across both individual and planetary health scales.

Future implementation of IAIEs may involve a continuous dynamically optimized cycle consisting of pathogen detection, computational prediction, targeted therapeutic selection, ecological monitoring, and iterative optimization. In such systems, artificial intelligence would function as a decision-support layer, while programmable therapeutics, microbiome interventions, and environmental surveillance would collectively provide real-time dynamically optimized responses to pathogen evolution. This framework extends beyond individual technologies and represents a new model of ecosystem-oriented antimicrobial medicine.

## 8 Conclusion

The accelerating global burden of antimicrobial resistance highlights the limitations of conventional antibiotic-centered strategies and underscores the need for innovative, contiously updated approaches to infectious disease management. Emerging technologies—including artificial intelligence-assisted antimicrobial discovery, programmable CRISPR-based therapeutics, engineered bacteriophages, antimicrobial peptides, microbiome-directed interventions, anti-virulence strategies, and nanotechnology-enabled delivery systems—have each demonstrated considerable potential to improve the precision and sustainability of antimicrobial therapy. However, each of these modalities also possesses important biological, technical, regulatory, and translational limitations. For example, antimicrobial peptides and anti-virulence therapeutics, although designed to reduce selective pressure under specific conditions, remain susceptible to evolutionary adaptation and therefore should not be regarded as inherently resistance-proof. The Intelligent Anti-Infective Ecosystem (IAIE) proposed in this Perspective should therefore be viewed not as a definitive solution to antimicrobial resistance, but as a conceptual systems framework that seeks to coordinate complementary therapeutic approaches through continuous integration of pathogen genomics, artificial intelligence-assisted prediction, microbiome dynamics, ecological monitoring, and longitudinal clinical feedback. The principal contribution of IAIE lies in its emphasis on dynamically optimized therapeutic coordination rather than the assumption that convergence alone can overcome the evolutionary capacity of microbial pathogens. Consequently, IAIE is proposed as a hypothesis-generating framework that may improve the adaptability and resilience of future antimicrobial strategies by integrating complementary technologies capable of responding to changing biological and ecological conditions. Whether such coordinated systems can meaningfully reduce resistance emergence, preserve microbiome integrity, and improve long-term clinical outcomes remains an important question for future multidisciplinary research. Addressing this challenge will require rigorous preclinical investigation, prospective clinical evaluation, standardized outcome measures, and continued collaboration across microbiology, infectious diseases, artificial intelligence, systems biology, synthetic biology, and One Health disciplines. Rather than advocating a single superior antimicrobial technology, this Perspective proposes that the next generation of antimicrobial innovation will depend increasingly on the intelligent coordination of complementary therapeutic strategies through data-driven, responsive and continuously learning systems. Whether such ecosystems improve clinical outcomes remains an open scientific question that now warrants systematic multidisciplinary investigation.

## Data Availability

The original contributions presented in the study are included in the article/supplementary material. Further inquiries can be directed to the corresponding author.
